# Assessing subcutaneous adipose tissue by simple and portable field instruments: Skinfolds versus A-mode ultrasound measurements

**DOI:** 10.1371/journal.pone.0205226

**Published:** 2018-11-29

**Authors:** Carla Pérez-Chirinos Buxadé, Toni Solà-Perez, Jorge Castizo-Olier, Marta Carrasco-Marginet, Alex Roy, Michael Marfell-Jones, Alfredo Irurtia

**Affiliations:** 1 INEFC-Barcelona Sports Sciences Research Group, Institut Nacional d’Educació Física de Catalunya (INEFC), University of Barcelona, Barcelona, Spain; 2 Department of Experimental Sciences and Technology, University of Vic, Vic, Spain; 3 Universidad Católica San Antonio de Murcia (UCAM), Murcia, Spain; University of Maiduguri College of Medical Sciences, NIGERIA

## Abstract

**Purpose:**

This study compared subcutaneous adipose tissue (SAT) measurements using a skinfold caliper and Renco Lean-Meater Series 12 A-mode portable ultrasound scanner (A-US). It aimed to assess their inter- and intra-rater reliability and measure the agreement between both methods.

**Methods:**

Eighty-four volunteers of different fitness levels were divided into three groups by Ʃ6 skinfolds: G1 ≤ 55 mm (n = 33 males); G2 > 55 mm (n = 32 males); G3 = 98.0 ± 52.3 mm (n = 19 females). Triceps, subscapular, biceps, iliac crest, supraspinal, abdominal, front thigh and medial calf were assessed by ultrasound and skinfolds. Two technicians for both tools performed triplicate measures. Intraclass correlation (ICC), technical error of measurement (TEM) and coefficients of variation (CVs) were applied for test-retest and inter-rater reliability. Non-Parametric statistics were used in order to establish possible statistical differences and correlation between skinfolds thickness and uncompressed subcutaneous adipose tissue thickness from ultrasound. The amount of agreement between both methods was assessed with Lin’s coefficient and a scatterplot of all site locations. A Bland-Altman plot was constructed to establish limits of agreement between groups and regression analysis was employed to assess the ability of skinfolds to explain the variance of ultrasound.

**Results:**

Test-retest ICC for skinfolds and ultrasound were higher than 0.989 and 0.793, respectively. Inter-rater ICC for skinfolds was 0.999 with a 95% CI of 0.995 to 0.999 and for ultrasound was 0.755 with a much larger 95% CI of 0.622 to 0.841. TEMs (> 8.50%) and CVs (> 6.72%) compromised ultrasound reliability. Statistical differences were found in most of the analysed anatomical sites (p < 0.001) except in biceps G2 (Z = -1.150, p = 0.25) and G3 (Z = -1.309, p = 0.19). Good correlations (r > 0.7, p ≤ 0.05) were reported at almost all anatomical sites and groups except for biceps (G1: Rho = 0.26, p = 0.140) and abdominal (G2: Rho = -0.16, p = 0.38; G3: Rho = 0.43, p = 0.068). Lin’s concordance correlation coefficient registered low values of agreement between skinfolds and A-mode ultrasound (ranged from—0.009–0.646). The scatterplot and the estimated regression line drawn through the midst of all anatomical sites of the whole sample had a slope of 0.51 and R^2^ adjusted = 0.62 was obtained. The combined analysis between the Bland-Altman plot and the linear regression showed that specifically in the G2 and G3 groups, as the SAT increases the differences between skinfolds and ultrasounds measurements also increases.

**Conclusions:**

The Renco Lean-Meater ultrasound is not interchangeable with skinfold measures. Its utility is questionable, particularly for assessing SAT in active adult populations. Its poor test-retest and inter-rater reliability as well as the lack of agreement when compared to the skinfolds would exclude the free use of the A-mode ultrasound scanner in its hypothetical replacing of the classical calipers.

## Introduction

Body composition is a key factor in determining health and sport performance [1–3]. Related to health, the amount and distribution of body fat is an important indicator of disease risk in both individuals and populations. The on-going epidemic of obesity (defined as BMI > 95th percentile) in children and adults has highlighted the significance of excess body fat amounts for short and long term health [4–6]. In regard to sport performance, monitoring body fat in athletes may assess the effectiveness of an exercise or dietary intervention as well as define a performance or selection criterion [3]. Furthermore, in weight-sensitive sports, it may be used to monitor the athletes’ health status because many of them aim for short- term body mass reduction or maintaining a very low weight in order to obtain a competitive advantage [7]. Nevertheless, sometimes these sudden changes may induce severe medical problems such as loss of tissue or insufficient bone mineral density [1,8,9].

Understanding that body fat has a large impact on health and performance optimisation leads us to assume the importance of the assessment of body composition with accuracy, precision and reliability methods [3]. Therefore, it is now recognized that quantifying human body composition has formed a central part of medical and exercise science research, highlighting the assessment of body fatness which has been a prime focus of attention [5,7,10].

The choice of body composition technique for the assessment of adipose tissue often depends on the intended purpose for which the data are to be used, as well as the available technology [3]. The reference and laboratory methods are the most accurate techniques for assessing body composition. However, they are the least used in both sports and health. Some of these limitations include [2]: lack of published normative data (e.g. multi-component models), radiation exposure (e.g. CT-imaging), time and financial costs (medical imaging, densitometry, photonic scanning, ultrasound…) and specialized personnel or training to operate (e.g. dual-energy x-ray absorptiometry).

Nowadays, it is very difficult to find small, portable, easy-to-use, economical and validated tools for body composition assessment in both sport and health [7,11]. One of the methods that is closest to meeting these parameters is ultrasound. Previous ultrasound research has used B-mode (brightness-mode) for diagnostic imaging at varying frequencies, from 3 to 22 MHz, in order to compare ultrasound with other methods by obtaining an image of subcutaneous fat [7,10,12–18]. However, the significant economic costs and the need for software that depends on interpretation by highly-specialized professionals have prevented the use of these devices in most contexts [19]. In recent years, novel and relatively-inexpensive A-mode (amplitude-mode) ultrasound devices have appeared which could provide a lower cost alternative to B-mode ultrasounds for assessing subcutaneous adipose tissue [20].

Unlike previous research, which has focused on measuring the estimation of %BF [20–24], this study compares SAT measurements in millimetres using a skinfold caliper and Renco Lean-Meater Series 12 A-mode portable ultrasound scanner. It aimed to assess the inter- and intra-rater reliability of both methods, particularly in the A-US and measure the amount of agreement between them.

## Materials and methods

### Subjects

One hundred and eleven volunteers including male (n = 80) and female (n = 31) were recruited for the study from January 2016 until April 2016. Twenty-seven (male, n = 15; female, n = 12) did not meet the inclusion criteria. Inclusion criteria were: (1) to be an adult (over 18 years old and under 70 years old); (2) to be an active person who has practiced at least half an hour of moderate-intensity physical activity on most days of the week [5] during the previous two years; (3) not to have performed physical exercise or ingested food in the previous three hours; (4) not to present injuries or any clinical condition at the time of the study; (5) not to have taken any medications; (6) to be measured by two technicians for both tools. Eighty-four subjects were selected (male, n = 65; female, n = 19). The study sample was classified into three groups, based on Ʃ6 skinfolds which represents SAT distribution in the whole body: *upper limbs (triceps and subscapular); trunk (supraspinal and abdominal); lower limb (front thigh and medial calf)*. G1 (males, Ʃ6 ≤ 55 mm) n = 33; G2 (males, Ʃ6 > 55 mm) n = 32 and G3 (females, Ʃ6 = 98.0 ± 52.3 mm) n = 19. The main characteristics of the volunteers are shown in Table 1. All subjects voluntarily participated in the study and signed a written informed consent. The study was conducted following the WMA Helsinki Declaration Statement [25] and approved by the Ethics Committee for Clinical Sport Research of Catalonia.

**Table 1:** Descriptive statistics of somatic characteristics (mean ± SD).

	G1(n = 33) males	G2 (n = 32) males	G3 (n = 19) females	All (n = 84)
Chronological age (years)	26.7 ± 3.9	37.0 ± 12.1	31.6 ± 14.5	31.7 ± 11.3
Height (cm)	181.0 ± 8.1	176.7 ± 6.6	163.2 ± 7.5	175.3 ± 10.1
BM (kg)	77.5 ± 7.5	78.0 ± 10.0	58.2 ± 7.5	73.3 ± 11.8
BMI (kg/m^2^)	23.6 ± 1.4	25.0 ± 2.9	22.0 ± 3.6	23.8 ± 2.8

BM, body mass; BMI, body mass index.

### Study design

This was a repeated-measures design. Eight variables were assessed for ultrasound and skinfolds at eight sites: triceps, subscapular, biceps, iliac crest, supraspinal, abdominal, front thigh and medial calf. Two experienced technicians took all of the skinfolds and ultrasound measurements separately and blinded to each other’s results in order to evaluate test-retest and inter-rater reliability. Each variable was measured in triplicate, non-consecutively and in a single session. From the three initial measurements, two, which were most distant, were selected and the mean was used in post hoc analysis.

### Procedures

#### Preliminary procedures

All the subjects were asked to get undressed to their underwear. Height and body mass were measured with a portable stadiometer (Holtain stadiometer, Holtain Limited, Crymych, UK: range: 600–2100 mm; resolution: 1 mm) and a medical scale (Seca 710, Seca Corp., Hamburg, DE: range: 0.05–200 kg; resolution: 0.05 kg), respectively. The anatomical sites (subscapular, triceps, biceps, iliac crest, supraspinal, abdominal, front thigh, and medial calf) were established according to the *International Working Group of Kinanthropometry*, described by Ross & Marfell-Jones [26] and adopted by the *International Society for the Advancement of Kinanthropometry* (ISAK) as the “skinfolds of the ISAK restrictive profile”. Two ISAK accredited technicians Level 3 (Technician 1) and Level 1 (Technician 2) took all of the skinfolds and ultrasound measurements. All data were registered in millimetres on a modified ISAK proforma.

#### Skinfolds

Firstly, the anatomical sites were localized by a metallic anthropometric tape (RealmetBcn anthropometric tape, RealmetBcn, Torelló, ES) and marked on the skin of the participants with an easily-washable dermographic pencil (Mitsubishi Corp, Tokyo, JP). Secondly, the skinfolds of the eight anatomical sites were taken with a calibrated caliper (Harpenden HSB-BI, Holtain Limited, Sussex, UK: range: 0–80 mm; resolution: 0.20 mm; pressure: 10 g/mm^2^; accuracy: 99%).

#### Ultrasound

Ultrasound measurements were conducted using the A-mode ultrasound scanner (Renco Lean-Meater Series 12, Renco Corp. Minneapolis, US: resolution: 1 mm; pressure: omitted; accuracy: ± 1 digit). The ultrasound device was applied exactly at the same anatomical sites as skinfolds, using an innocuous and odourless conducting gel (Anagel Ultrasound Gel, Anagel, Surbiton, UK) to facilitate the transducer head signal, minimize the possible different compressions and avoid air bubbles that inhibit transmission of ultrasound [27].

### Data analysis

Means and standard deviations were calculated for all variables, and sample distributions were tested for normality with the Shapiro-Wilk test. Data did not follow normal distribution. Test-retest reliability and inter-rater reliability were examined using intraclass correlation coefficient (ICC) with two-way mixed average measures model, technical error of measurement (TEM) and coefficient of variation (CV). In addition, the standard error of measurement (SEM) was calculated to obtain the minimal difference (MD), as well as their percentages (SEM %) and (MD %), respectively. Statistical differences between both methods at each of the measured sites were calculated by Wilcoxon Signed Ranks Test. The correlation between values in millimetres obtained by skinfolds thickness and ultrasound measurements was evaluated with Spearman correlation coefficients. For agreement evaluation between both methods, Lin’s coefficient and a scatterplot of all site locations were used. A combined analysis between Bland-Altman plot and regression lines was constructed to establish limits of agreement between skinfolds and ultrasound within the groups. A linear regression analysis and lines of best fit equations were expressed in order to assess the ability of skinfolds to explain the variance of the ultrasound. Precise *p* values were reported and *p* ≤ 0.05 was considered significant. All data were analysed using SPSS 22 (IBM Inc., New York, US).

## Results

Reliability results appear in Table 2. With regard to skinfolds, a high test-retest and inter-rater reliability was obtained. The values obtained in the ICC were very high, with the lowest reported value at 0.989 ICC (95% CI = 0.983–0.993) by Technician 2. The values obtained in the TEM and in the CV also show a high reliability. TEM values were lower than 5% in all anatomical sites with the exception of triceps (9.01%), biceps (6.96%) and supraspinal (6.03%) by Technician 2. CV was inferior to 7% in all anatomical sites. With the highest value reported by technician 2 at the point of the biceps (6.58%).

**Table 2:** Reliability statistics for eight anatomical sites assessed with skinfolds and ultrasound methods.

		TEST RETEST RELIABILITY												INTER-RATER RELIABILITY			
		SEM mm (%)		MD mm (%)		TEM_intra_ (%)		CV_intra_ (%)		ICC				TEM_inter_	CV_inter_	ICC	
		Tech. 1	Tech. 2	Tech. 1	Tech. 2	Tech. 1	Tech. 2	Tech. 1	Tech. 2	Tech.1		Tech. 2		(%)	(%)	r	CI 95%
										r	CI 95%	r	CI 95%				
Triceps	SF	0.13 (1.25)	0.66 (6.13)	0.37 (3.43)	1.82 (16.99)	2.80	9.01	2.66	3.47	1.00	1.00–1.00	0.99	0.98–0.99	4.74	2.22	1.00	0.99–1.00
	US	0.60 (7.77)	0.94 (12.91)	1.65 (21.54)	2.61 (35.75)	15.7	25.98	14.73	20.62	0.97	0.96–0.98	0.92	0.88–0.95	14.94	12.57	0.94	0.91–0.96
Subscapular	SF	0.14 (1.24)	0.19 (1.72)	0.38 (3.43)	0.53 (4.76)	2.57	3.61	2.05	3.04	1.00	1.00–1.00	1.00	1.00–1.00	2.68	2.01	1.00	1.00–1.00
	US	0.69 (10.35)	0.52 (7.99)	1.91 (28.70)	1.44 (22.19)	18.69	16.39	12.51	13.92	0.91	0.87–0.94	0.95	0.92–0.97	11.53	8.56	0.94	0.90–0.96
Biceps	SF	0.11 (2.20)	0.15 (2.97)	0.31 (6.16)	0.41 (8.22)	4.43	6.96	3.78	6.58	1.00	1.00–1.00	1.00	1.00–1.00	3.28	2.64	1.00	1.00–1.00
	US	0.63 (15.43)	1.39 (34.69)	1.74 (42.86)	3.86 (96.26)	25.78	47.73	20.22	20.15	0.93	0.90–0.96	0.79	0.68–0.87	30.38	21.53	0.87	0.80–0.91
Iliac crest	SF	0.23 (1.61)	0.21 (1.45)	0.64 (4.50)	0.57 (4.01)	2.90	3.88	1.85	3.84	1.00	1.00–1.00	1.00	1.00–1.00	2.36	1.72	1.00	1.00–1.00
	US	0.77 (9.12)	0.43 (5.05)	2.14 (25.23)	1.20 (14.05)	16.19	10.57	12.77	8.19	0.94	0.91–0.96	0.98	0.96–0.98	14.26	10.25	0.90	0.85–0.94
Supraspinal	SF	0.18 (1.85)	0.26 (2.65)	0.50 (5.15)	0.72 (7.39)	3.28	6.03	2.25	5.44	1.00	1.00–1.00	1.00	1.00–1.00	2.84	2.25	1.00	1.00–1.00
	US	1.06 (15.9)	1.24 (18.96)	2.93 (44.15)	3.44 (52.6)	24.85	28.18	15.99	13.22	0.88	0.81–0.92	0.80	0.70–0.87	17.13	10.17	0.90	0.84–0.93
Abdominal	SF	0.38 (2.31)	0.18 (1.06)	1.06 (6.38)	0.49 (2.93)	3.81	2.30	2.25	2.00	1.00	1.00–1.00	1.00	1.00–1.00	2.40	1.53	1.00	1.00–1.00
	US	1.34 (16.74)	0.77 (9.65)	3.72 (46.43)	2.14 (26.68)	26.44	17.13	15.25	10.76	0.88	0.81–0.92	0.96	0.93–0.97	27.43	13.99	0.76	0.62–0.84
Front thigh	SF	0.17 (1.13)	0.15 (0.99)	0.47 (3.14)	0.41 (2.71)	2.50	2.17	2.32	1.91	1.00	1.00–1.00	1.00	1.00–1.00	1.49	1.10	1.00	1.00–1.00
	US	0.50 (6.39)	0.66 (8.51)	1.38 (17.75)	1.82 (23.51)	12.16	14.99	8.43	9.57	0.97	0.95–0.98	0.95	0.93–0.97	8.50	6.72	0.97	0.96–0.98
Medial calf	SF	0.16 (1.86)	0.12 (1.49)	0.43 (5.15)	0.34 (4.08)	4.26	4.34	4.07	4.78	1.00	1.00–1.00	1.00	1.00–1.00	2.68	2.65	1.00	1.00–1.00
	US	0.58 (9.86)	0.99 (16.72)	1.62 (27.35)	2.73 (46.27)	17.81	24.85	16.09	11.47	0.95	0.93–0.97	0.86	0.79–0.91	11.42	9.09	0.96	0.94–0.98

SF, skinfolds; US, ultrasound; Tech., technician; SEM, standard error of measurement; MD, minimal difference; TEM, technical error of measurement; CV, coefficient of variation; ICC, intraclass correlation coefficient; CI, 95% confidence interval.

Regarding the A-mode ultrasound, despite having obtained high intra and inter ICC values, with the lowest reported value at 0.793 ICC (95% CI = 0.681–0.865) by Technician 2, the high values obtained in the TEM_intra_ (ranged from 10.57% to 47.73%), TEM_inter_ (8.50% - 30.38%), CV_intra_ (8.43% - 20.62%) and CV_inter_ (6.72% - 21.53%) questioning the test-retest and inter-rater reliability of the method.

The MDs for the skinfolds method were different at each site and ranged from 0.31mm (6.16%) at the biceps site to 1.06 mm (6.38%) at the abdominal site for Technician 1 and from 0.34 mm (4.08%) at medial calf site to 1.82 mm (16.99%) at triceps site for Technician 2. MDs at the same site changed when using ultrasound. Values ranged from 1.38 mm (17.75%) at the front thigh site to 3.72 mm (46.43%) at the abdominal site for Technician 1 and from 1.20 mm (14.05%) at the iliac crest site to 3.86 (96.26%) mm at the biceps site for Technician 2.

In Table 3, Statistical differences, correlations and linear regression analysis between skinfolds and ultrasound measurements are shown. All variables were higher when measuring with calipers in comparison to A-mode ultrasound scanner. Only the biceps did not register statistically-significant differences (G2: 5.2 ± 2.7 mm *vs*. 4.6 ± 1.5 mm; *p* = 0.25; G3: 7.8 ± 5.6 mm *vs*. 6.3 ± 2.3 mm; *p* = 0.19). All variables correlated well between both methods, except the biceps (G1: *Rho* = 0.26; *p* = 0.140) and the abdominal sites (G2: *Rho* = -0.16; *p* = 0.38; G3: *Rho* = 0.43; p = 0.068). Lin’s concordance correlation coefficient registered low values of agreement between skinfolds and A-mode ultrasound (ranged from—0.009–0.646) and these values were significant in G1 in subscapular (ρc = 0.054; 95% CI = -0.004–0.112) and biceps (ρc = 0.149; 95% CI = -0.018–0.308) sites; and in G2 in abdominal site (ρc = - 0.009; 95% CI = -0.054–0.036). Linear regression analysis confirmed these results with low levels of explanation of the model for the G1 in the subscapular (R^2^_adjusted_ = 0.09; *p* = 0.055), biceps (R^2^_adjusted_ = 0.07; *p* = 0.077), supraspinal (R^2^_adjusted_ = 0.26; *p* = 0.001) and medial calf (R^2^_adjusted_ = 0.27; *p* = 0.001) and especially in the abdomen site in the rest of the groups (G2: R^2^_adjusted_ = -0.03; *p* = 0.67; G3: R^2^_adjusted_ = 0.24; *p* = 0.020; All: R^2^_adjusted_ = 0.17; *p* = 0.001).

**Table 3:** Statistical differences, correlation, agreement and regression analysis between skinfolds and ultrasound measurements.

		Skinfolds	Ultrasound	Wilcoxon		Spearman		Lin's concordance		Linear regression				
				Z	p	Rho	p	ρc	CI 95%	Equation	F	p	R^2^_adjusted_	SEE
Triceps (mm)	G1	6.4 ± 1.6	5.4 ± 0.7	-3.950	0.001	0.64	0.001	0.39	0.21–0.54	y = 0.313x +3.416	27.081	0.001	0.45	0.53
	G2	12.0 ± 4.4	7.4 ± 1.2	-4.918	0.001	0.68	0.001	0.17	0.08–0.26	y = 0.187x +5.201	24.981	0.001	0.44	0.91
	G3	16.2 ± 8.7	11.2 ± 4.6	-3.260	0.001	0.79	0.001	0.58	0.36–0.73	y = 0.470x +3.625	58.413	0.001	0.76	2.26
	All	10.8 ± 6.3	7.5 ± 3.2	-7.308	0.001	0.83	0.001	0.58	0.49–0.66	y = 0.445x +2.715	255.429	0.001	0.75	1.59
Subscapular (mm)	G1	7.7 ± 1.1	5.2 ± 0.6	-5.013	0.001	0.34	0.053	0.05	-0.01–0.11	y = 0.189x + 3.752	3.986	0.055	0.09	0.58
	G2	13.8 ± 8.1	7.5 ± 1.9	-4.937	0.001	0.86	0.001	0.19	0.09–0.29	y = 0.162x + 5.330	27.063	0.001	0.46	1.41
	G3	12.4 ± 10.1	7.3 ± 2.8	-3.825	0.001	0.88	0.001	0.32	0.15–0.47	y = 0.214x + 4.686	22.891	0.001	0.55	1.91
	All	11.1 ± 7.4	6.6 ± 2.1	-7.962	0.001	0.79	0.001	0.30	0.22–0.37	y = 0.217x + 4.186	109.104	0.001	0.57	1.40
Biceps (mm)	G1	3.2 ± 0.6	2.2 ± 1.6	-2.905	0.004	0.26	0.140	0.15	-0.02–0.31	y = 0.858x - 0.559	3.335	0.077	0.07	1.59
	G2	5.2 ± 2.7	4.6 ± 1.5	-1.150	0.250	0.74	0.001	0.51	0.27–0.68	y = 0.350x + 2.773	18.491	0.001	0.36	1.21
	G3	7.8 ± 5.6	6.3 ± 2.3	-1.309	0.191	0.80	0.001	0.56	0.37–0.70	y = 0.349x + 3.621	40.992	0.001	0.69	1.29
	All	5.0 ± 3.6	4.0 ± 2.4	-3.227	0.001	0.79	0.001	0.65	0.53–0.74	y = 0.495x + 1.562	95.371	0.001	0.54	1.64
Iliac Crest (mm)	G1	9.1 ± 2.6	6.5 ± 1.3	-4.927	0.001	0.63	0.001	0.35	0.19–0.48	y = 0.396x + 2.903	45.054	0.001	0.58	0.87
	G2	18.3 ± 6.1	10.4 ± 2.2	-4.881	0.001	0.81	0.001	0.19	0.09–0.28	y = 0.265x + 5.600	33.969	0.001	0.52	1.55
	G3	16.2 ± 8.3	8.8 ± 3.0	-3.823	0.001	0.89	0.001	0.34	0.18–0.48	y = 0.337x + 3.359	144.516	0.001	0.89	0.98
	All	14.2 ± 7.0	8.5 ± 2.7	-7.877	0.001	0.90	0.001	0.38	0.30–0.45	y = 0.346x + 3.617	302.358	0.001	0.78	1.27
Supraspinal (mm)	G1	5.7 ± 1.1	4.8 ± 0.9	-3.853	0.001	0.35	0.045	0.37	0.14–0.57	y = 0.423x + 2.434	12.152	0.001	0.26	0.77
	G2	12.4 ± 6.2	7.8 ± 2.3	-4.806	0.001	0.79	0.001	0.38	0.24–0.50	y = 0.316x + 3.947	80.465	0.001	0.72	1.22
	G3	12.2 ± 8.7	7.5 ± 3.0	-3.401	0.001	0.90	0.001	0.43	0.25–0.57	y = 0.305x + 3.803	53.352	0.001	0.74	1.54
	All	9.7 ± 6.5	6.6 ± 2.5	-7.161	0.001	0.87	0.001	0.50	0.42–0.56	y = 0.344x + 3.263	291.805	0.001	0.78	1.18
Abdominal (mm)	G1	9.2 ± 2.3	6.1 ± 1.3	-5.013	0.001	0.79	0.001	0.27	0.14–0.39	y = 0.423x + 2.221	38.581	0.001	0.54	0.89
	G2	23.8 ± 9.0	8.6 ± 2.3	-4.918	0.001	-0.16	0.387	-0.01	-0.05–0.04	y = -0.018x + 9.070	0.156	0.696	-0.03	2.30
	G3	17.5 ± 9.1	10.3 ± 4.6	-3.421	0.001	0.43	0.068	0.29	0.03–0.49	y = 0.269x + 5.622	6.616	0.020	0.24	4.01
	All	16.7 ± 9.6	8.0 ± 3.2	-7.678	0.001	0.59	0.001	0.15	0.07–0.22	y = 0.140x + 5.704	18.066	0.001	0.17	2.88
Front thigh (mm)	G1	8.8 ± 2.2	5.6 ± 1.0	-4.977	0.001	0.65	0.001	0.19	0.09–0.28	y = 0.316x + 2.854	29.676	0.001	0.47	0.72
	G2	15.2 ± 3.6	8.0 ± 1.5	-4.937	0.001	0.73	0.001	0.11	0.04–0.17	y = 0.278x + 3.791	23.984	0.001	0.43	1.15
	G3	25.6 ± 11.0	11.1 ± 3.1	-3.824	0.001	0.83	0.001	0.17	0.07–0.27	y = 0.249x + 4.727	65.890	0.001	0.78	1.43
	All	15.0 ± 8.6	7.8 ± 2.8	-7.952	0.001	0.91	0.001	0.32	0.25–0.39	y = 0.294x + 3.347	435.936	0.001	0.84	1.11
Medial calf (mm)	G1	4.8 ± 1.0	4.2 ± 1.2	-2.902	0.004	0.57	0.001	0.46	0.19–0.67	y = 0.651x + 1.083	12.593	0.001	0.27	1.04
	G2	8.6 ± 2.7	6.2 ± 1.1	-4.871	0.001	0.78	0.001	0.35	0.21–0.47	y = 0.352x + 3.178	62.290	0.001	0.66	0.66
	G3	14.0 ± 8.1	8.4 ± 3.4	-3.824	0.001	0.93	0.001	0.48	0.31–0.63	y = 0.408x + 2.679	471.634	0.001	0.96	0.64
	All	8.3 ± 5.4	5.9 ± 2.5	-7.213	0.001	0.91	0.001	0.61	0.53–0.67	y = 0.426x + 2.368	616.483	0.001	0.88	0.85
Ʃ6 (mm)	G1	42.7 ± 5.4	31.4 ± 3.6	-5.012	0.001	0.66	0.001	0.15	0.07–0.24	y = 0.456x + 11.922	28.089	0.001	0.46	2.63
	G2	85.8 ± 28.1	45.6 ± 6.5	-4.937	0.001	0.72	0.001	0.11	0.05–0.18	y = 0.178x + 30.318	44.810	0.001	0.59	4.16
	G3	98.0 ± 52.3	55.8 ± 18.1	-3.823	0.001	0.92	0.001	0.36	0.19–0.50	y = 0.323x + 24.177	120.239	0.001	0.87	6.55
	All	71.6 ± 38.3	42.3 ± 13.6	-7.961	0.001	0.92	0.001	0.38	0.30–0.45	y = 0.323x + 19.176	401.254	0.001	0.83	5.64

G1: 33 males Ʃ6 ≤ 55 mm; G2: 32 males Ʃ6 > 55 mm; G3: 19 females; All: 84 males & females; Z, Wilcoxon signed ranks test; Rho, Spearman correlation; ρc, Lin's concordance; F, ANOVA statistic; R^2^_adjusted_, adjusted coefficient of determination; SEE, standard error of the estimate; CI, 95% confidence interval; p, level of significance (p≤0.05).

**Fig 1:**
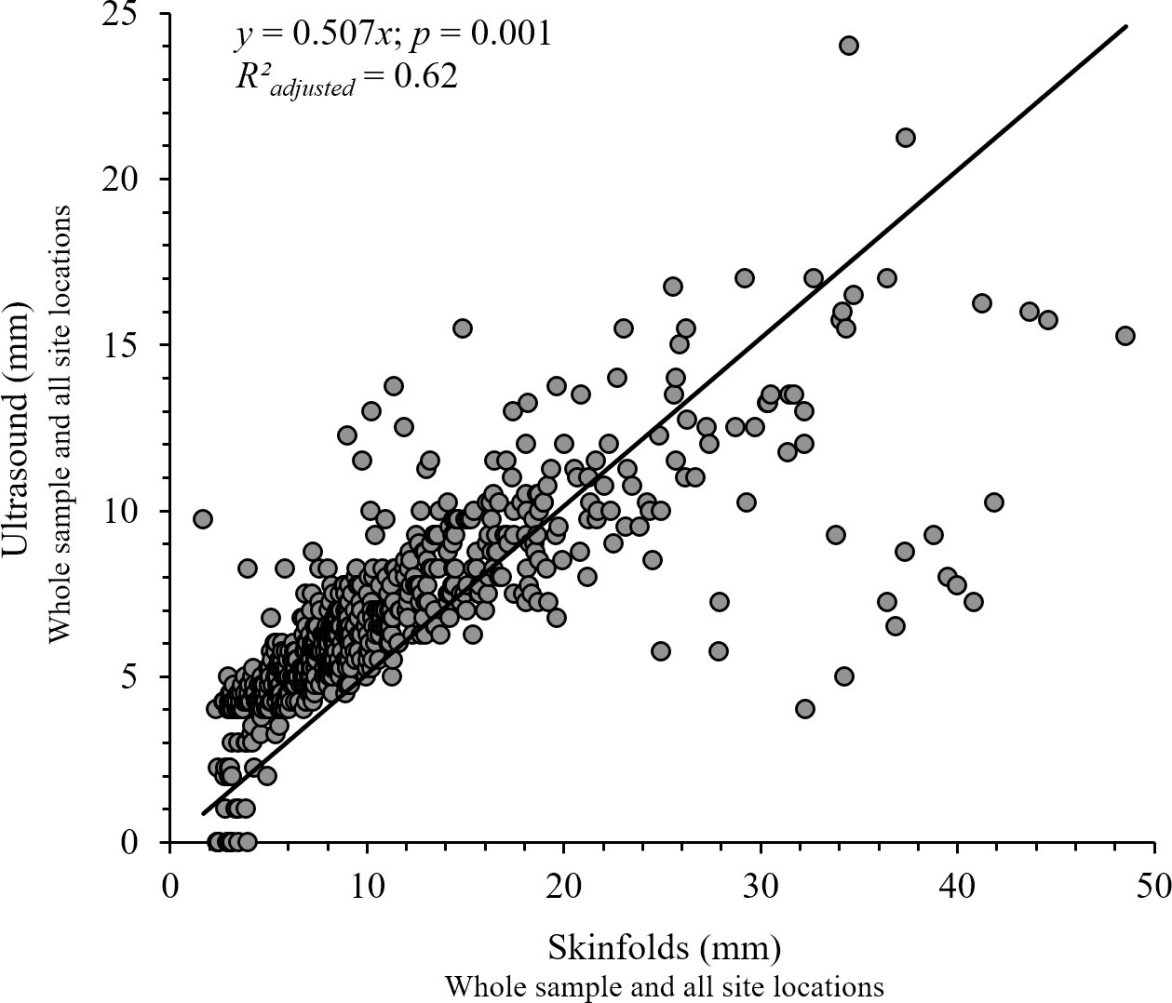
The Scatter Plot "Skinfolds, Ultrasound" and the line of best fit for all anatomical sites and all groups.

The scatterplot and the estimated regression line drawn through the midst of the points had a slope of 0.51 mm per mm and a value of R^2^_adjusted_ = 0.62 was obtained. Points scattered from the regression line and from the 45-degree line through the origin that would be obtained if there were perfect agreement when the skinfold thickness overpass 25 mm (Fig 1).

**Fig 2:**
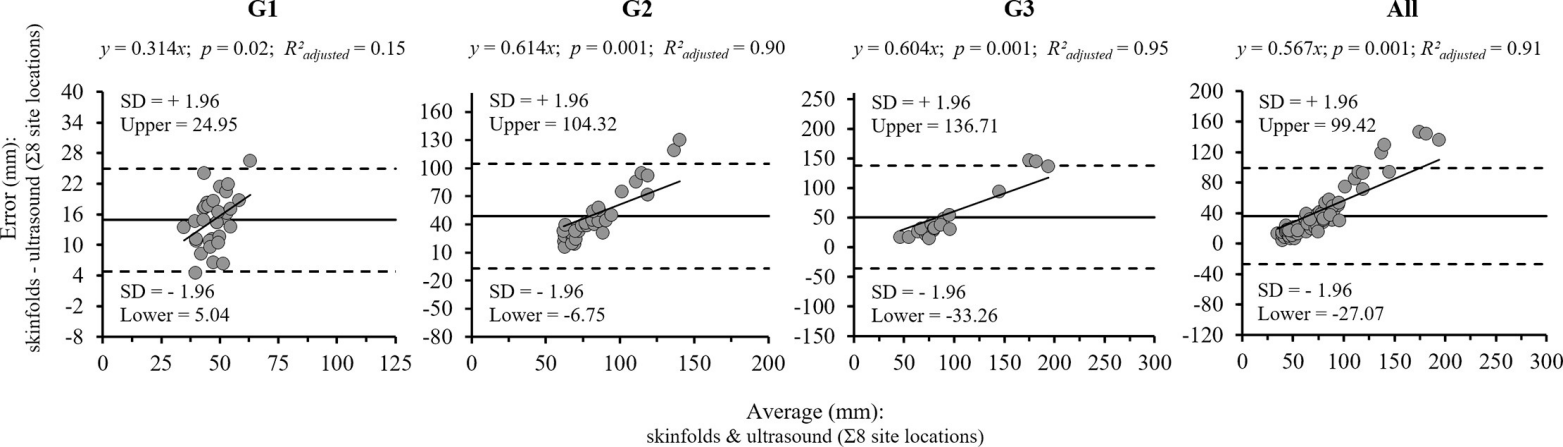
Bland-Altman plots and linear regression analysis for Ʃ8 anatomical sites (G1, G2, G3 and All).

The combined analysis between the Bland-Altman plot and the linear regression (Fig 2) showed that in the whole sample, and specifically in the G2 and G3 groups, as the SAT increased the differences between skinfolds and ultrasound measurements increased. This dependent relationship is explained with an explanatory power of 90.0% and 95.0%, respectively. Contrastingly, in the group with the lowest SAT (G1), there is no such relationship (R^2^_adjusted_ = 0.15).

## Discussion

The purpose of this study was to test the reliability and the degree of agreement of the A- mode portable ultrasound scanner (Renco Lean-Meater Series 12) for assessing subcutaneous adipose tissue in active adult population by comparing it to skinfolds. To our knowledge, this study used the largest and most varied sample to date to compare these two methods at anatomical-site locations.

One of the features of the present study was reporting the skinfolds thickness in millimeters as a valid measure thus avoiding the limitations of conventional %BF, since they are not affected by regression adjustments to estimate body composition [28]. Estimation of body fat drives additional errors of the reference method (usually densitometry), which have been identified to be greater amongst athletic groups [29].

Skinfolds measurement is a widely used, but admittedly not gold standard method. It is present in most body composition studies and shows high correlation with several techniques such as MRI [30], computed tomography [31], bioimpedance [21, 32] and ultrasound [18, 33]. Despite the main limitation of the compression and the disadvantage that it can be inaccurate in very obese people, it is considered sufficiently accurate for valid adipose tissue assessment [3,28,29]. Its widespread use is an important reason to compare another modality of measurement which would be easy to apply and equally as portable and competitive in cost.

A-mode portable ultrasounds have generally been designed for animal use, to determine the amount of fat mass in pigs [34] or the pregnancy status in fishes [35]. Nowadays, their relatively low cost, portable nature and the advantage of reporting the result directly in millimetres, are enhancing scientific interest in their applicability in humans. In this regard, accuracy and reliability have recently been tested in athletes [20,23], young men [36] and different population profiles such as overweight and obese people [22], and the military [21].

Although there are initiatives that try to standardize the anatomical points to place the US [7], there are no universally-accepted guidelines for their application and there is still a lack of standardization with regard to several aspects of ultrasound measurement (e.g., optimal scanning frequency and distance or length of scan). Toomey et al. [37] recently examined the technical aspects of using ultrasound to measure subcutaneous adipose tissue. They reported a reduced SAT thickness by a mean of 25–37% when the operator applied maximal force to the transducer. Müller et al. [7] confirm that adipose tissue compression was significantly reduced by using a thick layer of ultrasound gel (3–5 mm).

Regarding reliability, both technicians had high test-retest and inter-rater ICCs for both methods, which mean a high consistency when taking measures at each of the anatomical sites. Other studies have already reported similar results when evaluating intra- and inter-reliability for skinfolds [20, 38] and for A-mode ultrasound [22]. However, TEMs and CVs are concordant with the ICC only in the case of skinfolds. Therefore, intra- and inter-reliability of the ultrasound has been compromised.

The minimal differences (MD) which needed to be considered as real changes were calculated for each anatomical site and for both methods. Generally, higher values of MD were those from ultrasound measurements (< 4mm), while MD for skinfolds were < 2mm. Wagner et al. [20] described similar findings, but reported changes in %BF. However, for the same site, the value was different depending on the technician (Table 2).

Another finding concerned the relationship between skinfolds and ultrasound at the individual measurement sites (Table 3) and (Fig 1). We found all variables to be higher when measuring with calipers in comparison to A-mode ultrasound scanner. These results are consistent with those obtained in other studies [20, 39] and are expected because a skinfold involves a double layer of skin along with the compressed fold of subcutaneous fat [40, 41] whereas the ultrasound method is directly measuring the subcutaneous fat thickness [11]. Despite the difference in absolute value, it is logical to assume a high correlation between methods because they are both measuring subcutaneous fat. In the present study, correlation coefficients ranged between 0.34–0.93 with a p ≤ 0.05 at nearly every site for three groups: G1 (Rho = 0.34–0.79; *p* ≤ 0.05) with highest correlations found in abdominal and front thigh sites, and lowest correlations found in subscapular and supraspinal sites;G2 (Rho = 0.68–0.86; *p* ≤ 0.05) with highest correlations found in subscapular and iliac crest sites, and lowest correlations assumed in triceps and front thigh sites; and G3 (Rho = 0.80–0.93; *p* ≤ 0.05) with the highest correlations found in medial calf and supraspinal sites, and the lowest correlations assumed in biceps and triceps sites. Similar results to these for G1 and G2 were reported in previous studies [20] and [21], respectively. Considering the triceps site of our 19 females (G3), the correlation (Rho = 0.80) between both methods was very similar to the correlation found in 23 Division-I female athletes from the *National Collegiate Athletic Association* (NCAA) (r = 0.82) [20]. Other authors have reported high correlations in subcutaneous fat measurements when comparing skinfolds and ultrasound methods in adult populations from 16 to 87 years [32, 33, 42].

Concerning agreement, Lin’s coefficient is 1 when all the points lie exactly on the 45-degree line which means a perfect agreement, but we found this was did not occur. Independently of the group and the anatomical sites, a sufficient degree of agreement was not found to consider both methods interchangeable.

Similar results were found in scatterplot and the estimated regression line drawn through the midst of the points. The reported slope was far from the slope of 1 mm per mm for the 45-degree line through the origin corresponding to perfect agreement. It was shown that when the skinfold thickness exceeded 25 mm, points scattered from the 45-degree line. The majority of these points corresponded to the abdominal site. The greatest agreement between both methods was obtained for skinfold thickness from 10 to 20 mm. However, even in this range, some points moved away from the line. Adjusted coefficient of determination indicated that approximately 60% of the variation in A-mode ultrasound could be explained by its linear relationship with the skinfold method—a low percentage of agreement.

All in all, we located two anatomical sites that did not mirror the same statistical behaviour as the other six, particularly in some of the analysed groups. These were the biceps and the abdominal sites. Referring to the biceps site in G1, no significant correlation nor concordance nor linearity were found between skinfolds compared to ultrasound measures (Table 3). It might be explained as a result of the A-mode ultrasound range of measurement, established from 4 to 35 mm. Most of G1 participants had fewer values than 4 mm of SAT in the biceps site, and ultrasound did not detect them. This becomes clearly visible by looking at ordinate axis from Scatter Plot "Skinfolds, Ultrasound" (Fig 1). Concerning the abdominal site, it was the measure which had the lowest correlation between measurements of skinfold and ultrasound when unifying the three groups (Rho = 0.59). There was no significant correlation in G2 and G3. And in G2 no concordance was obtained nor was a linear relationship established between both methods. Scatter Plot "Skinfolds, Ultrasound" (Fig 1) also showed that as the thickness of the skinfolds increases (> 25 mm), the linear association between the methods decreases and therefore, in a linear model, one method is not explanatory of the other. It has been found that most points that move away from the 45-degree line correspond to abdominal sites, when SAT exceeds 25 mm. The difficulty in obtaining skinfolds where there is a greater amount of fat thickness [43] or the apparently complex structure from adipose tissue in the lower abdomen with the presence of an intermediate fascia structure (Camper’s fascia) could have affected our results [7].

Bland-Altman plots and linear regression analysis demonstrated that methods were not measuring the same anatomical unit (skinfolds: two lipid layers, ultrasound: a lipid layer). It was observed therefore that, the higher the SAT average of both instruments (approximately values from 100 mm), the theoretical ratio of 2:1 (SF:US) was lost, in this case increasing because of the greater increase registered in SF compared to the US. In the G1 group, however, the range of SAT values recorded from the average of both methods does not exceed 70 mm, a circumstance that might explain the absence of the large differences observed in groups G2, G3 and the global sample. This fact could be explained because the use of skinfold measurements is more difficult in overweight and obese subjects [43] and therefore its accuracy is compromised [5].

In summary, A mode Ultrasound is not interchangeable with SF. The utility of this US method is questionable, particularly for assessing SAT in active adult populations. Its poor test-retest and inter-rater reliability as well as the lack of agreement when compared to the SF would exclude the free use of the A-mode ultrasound scanner Renco Lean-Meater Series 12 as a replacement for the classical calipers.

Since the method used as a reference (SF) is not a gold standard, we cannot affirm or deny its accuracy and validity. More research, such as a multi-component validation study, which included sophisticated reference methods like nuclear magnetic resonance or dual-energy x-ray absorptiometry or a comparison with a validated B-mode ultrasound, is needed before this A-mode ultrasound device can be validated for use in the assessment of subcutaneous adipose tissue in humans.

### Practical applications

The search for new methods to evaluate subcutaneous adipose tissue and body composition in terms of health and performance provides new opportunities for the advance of kinanthropometry. This study tried to demonstrate the reliability of an alternative method to evaluate subcutaneous adipose tissue in humans. The main features of this A-mode ultrasound including its small size, portability, ease of use, the fact that it does not involve radiation and is relatively inexpensive give it many advantages over other imaging devices and laboratory methods to assess body composition.

Additionally, the ability to assess regional composition reporting the measure of subcutaneous adipose tissue direct in millimeters provides another advantage over many other methods and allows for unique assessments of some clinical populations. Nevertheless, the lack of reliability and accuracy when comparing it with skinfold measurements suggests that further testing is needed before it can be reliably used for this purpose.

## Supporting information

S1 Dataset Study database. (XLSX)
